# Impact of Chronic Kidney Disease on Use of Evidence-Based Therapy in Stable Coronary Artery Disease: A Prospective Analysis of 22,272 Patients

**DOI:** 10.1371/journal.pone.0102335

**Published:** 2014-07-22

**Authors:** Paul R. Kalra, Xavier García-Moll, José Zamorano, Philip A. Kalra, Kim M. Fox, Ian Ford, Roberto Ferrari, Jean-Claude Tardif, Michal Tendera, Nicola Greenlaw, Ph. Gabriel Steg for the CLARIFY Investigators

**Affiliations:** 1 Portsmouth Hospitals NHS Trust, Portsmouth, United Kingdom; 2 NHLI Imperial College, ICMS, London, United Kingdom; 3 Unitat Hospitalització, Servei de Cardiologia, Hospital de la Santa Creu i Sant Pau, Barcelona, Spain; 4 University Hospital Ramón y Cajal, Madrid, Spain; 5 Salford Royal NHS Foundation Trust, Salford, United Kingdom; 6 Royal Brompton Hospital, London, United Kingdom; 7 University of Glasgow, Glasgow, United Kingdom; 8 Department of Cardiology, Azienda Ospedaliero-Universitaria di Ferrara, Ospedale di Cona, Cona, Italy; 9 Montreal Heart Institute, Université de Montreal, Montreal, Canada; 10 Medical University of Silesia, Katowice, Poland; 11 Université Paris-Diderot, Sorbonne-Paris Cité, Paris, France; 12 INSERM U-1148, Paris, France; 13 Département Hospitalo-Universitaire FIRE, Hôpital Bichat, Assistance Publique – Hôpitaux de Paris, Paris, France; University Medical Center Utrecht, Netherlands

## Abstract

**Purpose:**

To assess the frequency of chronic kidney disease (CKD), define the associated demographics, and evaluate its association with use of evidence-based drug therapy in a contemporary global study of patients with stable coronary artery disease.

**Methods:**

22,272 patients from the ProspeCtive observational LongitudinAl RegIstry oF patients with stable coronary arterY disease (CLARIFY) were included. Baseline estimated glomerular filtration rate (eGFR) was calculated (CKD-Epidemiology Collaboration formula) and patients categorised according to CKD stage: >89, 60–89, 45–59 and <45 mL/min/1.73 m^2^.

**Results:**

Mean (SD) age was 63.9±10.4 years, 77.3% were male, 61.8% had a history of myocardial infarction, 71.9% hypertension, 30.4% diabetes and 75.4% dyslipidaemia. Chronic kidney disease (eGFR<60 mL/min/1.73 m^2^) was seen in 22.1% of the cohort (6.9% with eGFR<45 mL/min/1.73 m^2^); lower eGFR was associated with increasing age, female sex, cardiovascular risk factors, overt vascular disease, other comorbidities and higher systolic but lower diastolic blood pressure. High use of secondary prevention was seen across all CKD stages (overall 93.4% lipid-lowering drugs, 95.3% antiplatelets, 75.9% beta-blockers). The proportion of patients taking statins was lower in patients with CKD. Antiplatelet use was significantly lower in patients with CKD whereas oral anticoagulant use was higher. Angiotensin-converting enzyme inhibitor use was lower (52.0% overall) and inversely related to declining eGFR, whereas angiotensin-receptor blockers were more frequently prescribed in patients with reduced eGFR.

**Conclusions:**

Chronic kidney disease is common in patients with stable coronary artery disease and is associated with comorbidities. Whilst use of individual evidence-based medications for secondary prevention was high across all CKD categories, there remains an opportunity to improve the proportion who take all three classes of preventive therapies. Angiotensin-converting enzyme inhibitors were used less frequently in lower eGRF categories. Surprisingly the reverse was seen for angiotensin-receptor blockers. Further evaluation is required to fully understand these associations. The CLARIFY (ProspeCtive observational LongitudinAl RegIstry oF patients with stable coronary arterY disease) Registry is registered in the ISRCTN registry of clinical trials with the number ISRCTN43070564. http://www.controlled-trials.com/ISRCTN43070564.

## Introduction

Chronic kidney disease (CKD) is a powerful independent predictor of adverse prognosis following myocardial infarction (MI) [1], [2] or coronary revascularization [3], [4]. A recent study has shown that post MI the presence of CKD (defined as estimated glomerular filtration rate, eGFR, <60 mL/min/1.73 m^2^) was a stronger predictor of all-cause mortality than either a history of MI or diabetes [2]. Compared with a reference population without a history of MI, CKD or diabetes, the presence of CKD was associated with a 3.6-fold unadjusted relative rate of all-cause mortality; the respective rates for patients with history of MI or diabetes were 2.7 and 1.9.

A number of plausible reasons might explain this link. CKD may merely represent a bystander marker of advancing age and comorbidities. Alternatively pathophysiological derangements in patients with CKD such as activation of the renin-angiotensin-aldosterone systems, inflammatory immune activation or disordered calcium-phosphate metabolism might contribute to cardiovascular disease progression or expression [5], [6]. Underutilization of evidence-based treatments in patients with CKD and coronary artery disease (CAD) may also play a role. Historical data have suggested that despite major advances in secondary prevention following MI, patients with CKD are less commonly prescribed prognostically beneficial drugs. For example, data from 14,527 patients with acute MI complicated by heart failure (Valsartan in Acute Myocardial Infarction Trial) showed that declining eGFR was associated with increased risk of death and non-fatal cardiovascular outcomes [1]. Whilst patients with eGFR <45 mL/min/1.73 m^2^ were at highest risk of events, the use of aspirin, beta-blockers, statins or coronary revascularization was lowest in this group. A retrospective cohort study of Medicare patients with acute MI showed that those with CKD stage 4 (eGFR 15–29 mL/min/1.73 m^2^) were infrequently prescribed aspirin with beta-blockers (27.1%) and fewer than one in 10 were prescribed the combination of aspirin, beta-blockers and angiotensin-converting enzyme (ACE) inhibitors [7]. Similar data were found in a single-centre prospective study for patients discharged after acute MI [8].

Patients with CKD and CAD are at higher absolute risk of adverse events and many are therefore likely to derive marked benefit from secondary prevention. Identifying and subsequently rectifying deficiencies of care in such patients has the potential to impact positively on outcomes. The aims of the current study were to assess the frequency of CKD, define the associated demographics, and evaluate the impact of CKD on medical therapy in a large contemporary global study of patients with stable CAD: the ProspeCtive observational LongitudinAl RegIstry oF patients with stable coronary arterY disease (CLARIFY) [9]. We hypothesized that patients with advanced CKD would receive suboptimal secondary prevention compared to patients with preserved renal function. This unique, contemporary cohort study has enabled us to evaluate these objectives in detail.

## Methods

### Ethics Statement

The study was conducted in accordance with the principles in the Declaration of Helsinki and local ethical approval was obtained as necessary in all countries prior to recruitment. All patients gave written informed consent.

### Study Design

CLARIFY is an ongoing international, prospective, observational, longitudinal cohort study in outpatients with stable CAD. The study rationale and methods have been published elsewhere (further information can be found online at www.clarify-registry.com) [9], [10]. Briefly, the registry was designed to collect data on the current status of outpatients with stable CAD, including their demographic characteristics, clinical profiles, therapeutic strategies, and outcomes. More than 33,000 patients were enrolled in 45 countries in Africa, Asia, Australia, Europe, the Middle East, and North, Central and South America. A detailed list of countries, sites, and investigators has already been published [10]. Patients are followed up for 5 years with data collected prospectively at annual visits, and interim phone calls every 6 months. Patients are treated according to usual clinical practice at each institution, with no specific tests or therapies defined in the study protocol. The current study relates to data collected at baseline assessment.

### Study Design and Patients

The 2,884 participating physicians were selected on the basis of their geographic distribution; each was requested to recruit 10–15 consecutive stable outpatients with CAD to meet a predefined country target of 25 patients per million inhabitants (range 12.5–50) and obtain an epidemiologically representative population in each country. Eligible patients had stable CAD proven by a history of at least one of the following: documented MI (>3 months before enrolment); angiographic demonstration of coronary stenosis >50%; chest pain with evidence of myocardial ischaemia (stress electrocardiogram, stress echocardiograph or myocardial perfusion imaging); or coronary artery bypass graft (CABG) or percutaneous coronary intervention (PCI) performed >3 months before enrolment. These criteria were not mutually exclusive. Exclusion criteria included hospital admission for cardiovascular reasons (including revascularization) in the past 3 months, planned revascularization, or conditions hampering participation for the 5-year follow-up, such as limited cooperation, inability to provide informed consent, serious non-cardiovascular disease or conditions interfering with life expectancy (e.g. cancer, drug abuse) or severe other cardiovascular disease (e.g. advanced heart failure, severe valve disease, history of valve repair/replacement). CKD *per se* was not an exclusion criterion. To ensure that the study population was representative of stable CAD outpatients, recruitment of sites and subjects was based on predefined selection of physicians (cardiologists, as well as office-based primary care physicians and physicians based in hospitals with outpatient clinics) by national coordinators, using the best available epidemiological data in each country reflecting the burden of CAD; this was done in an attempt to provide a distribution of physicians across regions and locations (i.e. urban, suburban, or rural areas) mimicking the epidemiological patterns in each country. In each practice, patient enrolment was restricted over a brief period to achieve near consecutive patient enrolment in order to avoid selection bias. The first patient was included on 26 November 2009 and recruitment was completed on 30 June 2010.

### Data Collection

The investigators completed standardized electronic case report forms (eCRFs) at baseline. All forms were sent electronically to the data management centre where checks for completeness, internal consistency and accuracy were run. A number of measures were implemented to ensure data quality, including onsite monitoring visits of all data in randomly selected centres (1% per annum). Data quality control happened at face-to-face quality control visits involving review of source documents supporting the adequacy and accuracy of data collected on the case report forms. At baseline data were collected on demographics, medical history, risk factors and lifestyle, physical condition and vital signs, current symptoms and treatments. Available results of laboratory tests, invasive and non-invasive tests were collected, but no test was mandated by the study and there was no standardized measurement of the left ventricular ejection fraction.

Patient confidentiality was ensured by utilizing patient identification code numbers to correspond to the computer files. The study is registered (ISRCTN43070564).

### Statistical Analysis

All CLARIFY data are collected and analysed at an independent academic statistics centre at the Robertson Centre for Biostatistics, University of Glasgow, United Kingdom, which is responsible for the management of the database, all analyses (using the SAS statistical program, version 9.2 or higher), and storing the data according to regulations. Baseline characteristics for the whole population, according to renal function, are presented using descriptive statistics with mean (standard deviation [SD]) or median (quartile 1, quartile 3 [Q1, Q3]) for continuous variables, depending on the distribution of the data, and as counts (percentages) for categorical data.

We analysed the CLARIFY population at baseline grouped by eGFR according to the CKD-Epidemiology Collaboration (CKD-EPI) formula [11] (Table S2). Patients were categorized according to CKD stage [12]: stage 1, >89 mL/min/1.73 m^2^; stage 2, 60–89 mL/min/1.73 m^2^; stage 3a, 45–59 mL/min/1.73 m^2^; and stage 3b–5: <45 mL/min/1.73 m^2^. CKD-EPI was selected since data suggest that it may be more accurate than other creatinine-based formulae when applied to a population of patients in which a large proportion have eGFR>60 mL/min/1.73 m^2^ [11]. Patients with CKD stages 3b, 4 and 5 (eGFR<45 mL/min/1.73 m^2^) were grouped since it was anticipated that there would be small numbers with stages 4 or 5 in particular. The prevalence of renal dysfunction and the differential characteristics of these patients are described. Management of these patients is also depicted.

In order to explore medication use further, an analysis of how many patients received all cardiovascular preventive measures was performed. Data for each renal function group were analysed according to the number (%) of patients who received ‘all secondary preventive measures’, defined as taking at least one medication from all three of the following drug categories: (i) antiplatelet, (ii) statin, and (iii) ACE inhibitor or angiotensin receptor blocker (ARB).

Comparisons between the renal function groups were made using either one-way analysis of variance or the Kruskal–Wallis test for continuous data, depending on the distribution of the data, or the chi-square test for categorical data.

Univariate and multivariable logistic regression analyses were also performed to ascertain the relationship between renal dysfunction and management. Analyses were performed to examine the effect of renal dysfunction on the use of ACE inhibitors and then separately the use of ARBs. The multivariable analyses were adjusted for other clinical and demographic variables known to have a relationship with the use of these therapies, and included the following: age, body mass index (BMI), systolic blood pressure, diastolic blood pressure, gender, heart rate, smoking status, history of heart failure, angina, diabetes mellitus and hypertension.

## Results

**Figure 1:**
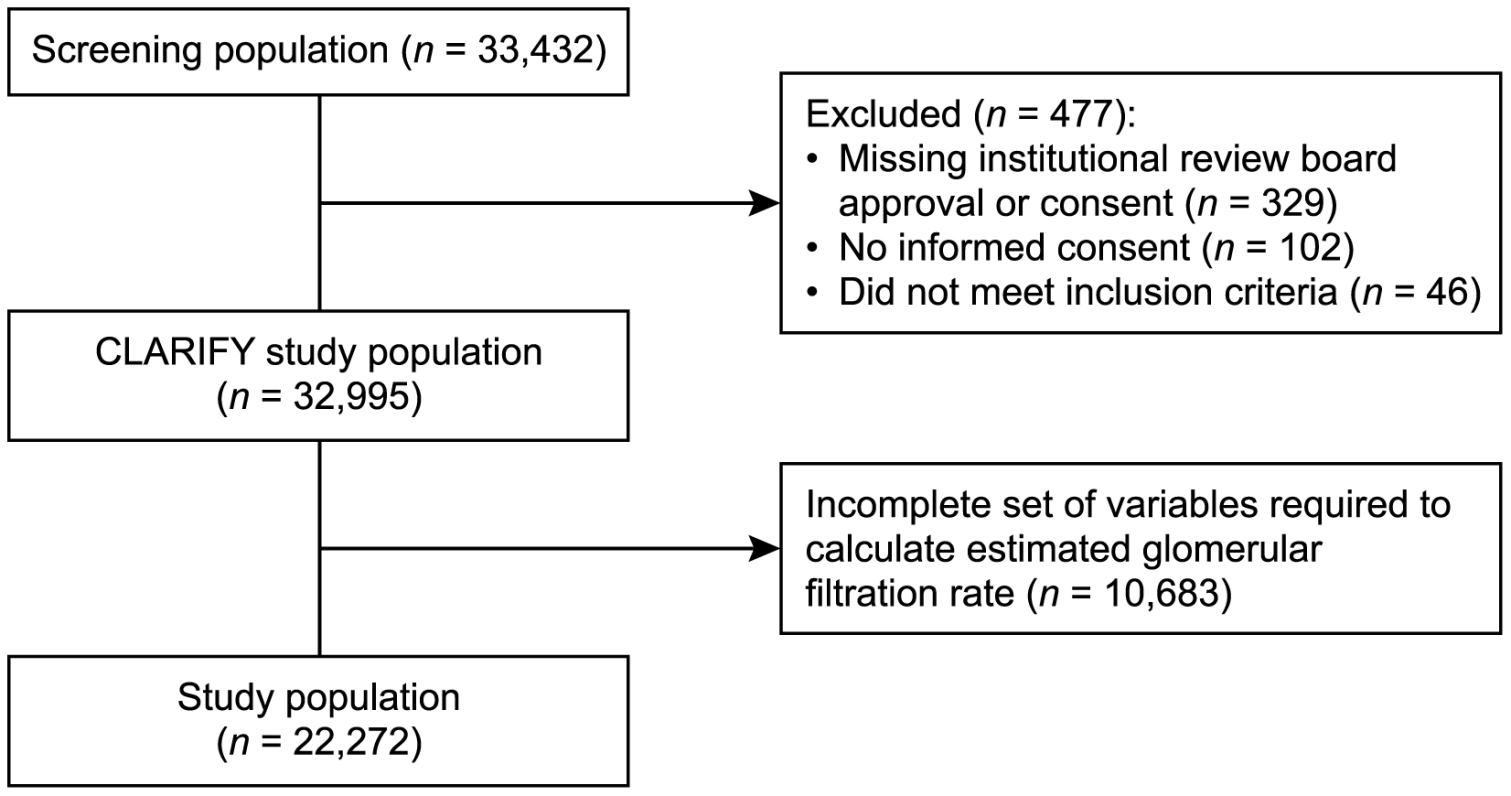
Patient flow chart.

Of the 33,432 patients screened, 329 patients were missing institutional review board approval or consent, 102 did not provide consent and 46 did not meet the inclusion criteria, leaving 32,955 patients for analysis. Of these, 22,272 patients had all of the baseline variables available to permit eGFR calculation and thereby formed the final study group (Figure 1). The mean age was 63.9±10.4 years, 77.3% were male, 61.8% had a history of MI, 71.9% had treated hypertension, 30.4% were diabetic, 75.4% were dyslipidaemic and 5.0% had a prior hospitalization for chronic heart failure (Table 1). The majority of patients were white (74.3%). Just over half of the study population had received PCI (56.6%) and 24.2% CABG; most (76.0%) were free of symptomatic angina.

**Table 1:** Demographic data: characteristics of the study population classified according to eGFR group calculated using the CKD-EPI.

Variable	Total (*n* = 22,272)	eGFR (mL/min/1.73 m^2^)				*p*-value^a^
		<45 (*n* = 1,528)	45–59 (*n* = 3,389)	60–89 (*n* = 11,826)	≥90 (*n* = 5,529)	
Age, mean (SD), years	63.9±10.4	71.4±9.5	69.8±8.8	64.7±9.5	56.5±8.8	<0.0001
Men, *n* (%)	17,221 (77.3)	968 (63.4)	2,283 (67.4)	9,331 (78.9)	4,639 (83.9)	<0.0001
BMI, median (Q1, Q3), kg/m^2^	27.5 (24.9, 30.5)	27.5 (24.7, 30.8)	27.5 (24.9, 30.7)	27.4 (25.0, 30.4)	27.5 (24.9, 30.8)	0.25
Time since 1st CAD diagnosis, median (Q1, Q3), years	4 (2, 9)	6 (2, 12)	6 (2, 11)	5 (2, 10)	3 (1, 7)	<0.0001
Myocardial infarction, *n* (%)	13,759 (61.8)	956 (62.6)	2,015 (59.5)	7,255 (61.4)	3,533 (63.9)	0.0002
PCI, *n* (%)	12,606 (56.6)	755 (49.4)	1,729 (51.0)	6,643 (56.2)	3,479 (62.9)	<0.0001
CABG, n (%)	5,386 (24.2)	462 (30.3)	977 (28.8)	2,901 (24.5)	1,046 (18.9)	<0.0001
Internal cardiac defibrillator, *n* (%)	284 (1.3)	54 (3.5)	56 (1.7)	132 (1.1)	42 (0.8)	<0.0001
Pacemaker, n (%)	489 (2.2)	88 (5.8)	117 (3.5)	237 (2.0)	47 (0.9)	<0.0001
Stroke, *n* (%)	951 (4.3)	137 (9.0)	205 (6.0)	484 (4.1)	125 (2.3)	<0.0001
Transient ischaemic attack, *n* (%)	694 (3.1)	92 (6.0)	178 (5.3)	311 (2.6)	113 (2.0)	<0.0001
Peripheral artery disease, *n* (%)	2,122 (9.5)	240 (15.7)	420 (12.4)	1,075 (9.1)	387 (7.0)	<0.0001
Hospitalization for CHF, *n* (%)	1,120 (5.0)	202 (13.2)	237 (7.0)	504 (4.3)	177 (3.2)	<0.0001
Treated hypertension, *n* (%)	16,000 (71.9)	1,297 (84.9)	2,660 (78.5)	8,423 (71.2)	3,620 (65.5)	<0.0001
Diabetes, *n* (%)	6,763 (30.4)	688 (45.0)	1,159 (34.2)	3,276 (27.7)	1,640 (29.7)	<0.0001
Dyslipidaemia, n (%)	16,794 (75.4)	1,189 (77.8)	2,542 (75.0)	8,969 (75.9)	4,094 (74.1)	0.009
Atrial fibrillation/flutter, *n* (%)	1,615 (7.3)	211 (13.8)	384 (11.3)	839 (7.1)	181 (3.3)	<0.0001
Asthma/COPD, *n* (%)	1,750 (7.9)	156 (10.2)	300 (8.9)	964 (8.2)	330 (6.0)	<0.0001
Smoking status, *n* (%)						
Current	2,828 (12.7)	99 (6.5)	254 (7.5)	1,436 (12.2)	1,039 (18.8)	
Former	10,302 (46.3)	669 (43.8)	1,479 (43.6)	5,619 (47.5)	2,535 (45.9)	
Never	9,137 (41.0)	760 (49.7)	1,656 (48.9)	4,768 (40.3)	1,953 (35.3)	
Angina and CCS class, *n* (%)						<0.0001
No Angina	1,6921 (76.0)	1,142 (74.7)	2,512 (74.1)	8,961 (75.8)	4,306 (77.9)	
CCS class I	1,527 (6.9)	98 (6.4)	222 (6.6)	832 (7.0)	375 (6.8)	
CCS class II	2,802 (12.6)	193 (12.6)	498 (14.7)	1,486 (12.6)	625 (11.3)	
CCS class III	953 (4.3)	90 (5.9)	151 (4.5)	506 (4.3)	206 (3.7)	
CCS class IV	61 (0.3)	5 (0.3)	6 (0.2)	35 (0.3)	15 (0.3)	

Abbreviations: CABG, coronary artery bypass graft; CAD, coronary artery disease; CCS, Canadian Cardiovascular Society; CHF, chronic heart failure; CKD-EPI, Chronic Kidney Disease–Epidemiology Collaboration; COPD, chronic obstructive pulmonary disease; eGFR, estimated glomerular filtration rate; NYHA, New York Heart Association; PCI, percutaneous coronary intervention; SD, standard deviation.

a *p*-value tests for differences across eGFR groups.

Table 1 also shows the demographics of patients by eGFR status. Chronic kidney disease (eGFR<60 mL/min/1.73^2^) was seen in 22.1%. Patients with lower eGFR were older, with a higher prevalence of women, comorbidities, cardiovascular risk factors and overt vascular disease. Patients with CKD were more likely to have undergone CABG but less likely to have had PCI compared to those with preserved renal function. The prevalence of atrial fibrillation/flutter (AF) and chronic heart failure increased in stepwise fashion whereas the proportion of active smokers decreased in patients with lower eGFR.

Clinical findings and investigations according to eGFR category are shown in Table 2. Patients with lower eGFR exhibited higher systolic blood pressures whilst the converse was seen for diastolic pressure. There were no clinically meaningful differences in heart rate or lipid profile. Haemoglobin concentration was progressively lower according to worse CKD class.

**Table 2:** Clinical finding and investigations of the study population classified according to eGFR group calculated using the CKD-EPI.

Variable	Total available	Total (*n* = 22,560)	eGFR (mL/min/1.73 m^2^)				*p*-value^a^
			<45 (*n* = 1,528)	45–59 (*n* = 3,389)	60–89 (*n* = 11,826)	≥90 (*n* = 5,529)	
Heart rate palpation, mean (SD), beats per min	22,269	68.5±10.7	69.4±11.4	68.6±11.1	68.0±10.6	69.1±10.5	<0.0001
Systolic BP, mean (SD), mm Hg	22,268	131.1±16.9	132.8±18.4	132.6±17.5	131.2±16.7	129.5±16.4	<0.0001
Diastolic BP, mean (SD), mm Hg	22,268	77.3±10.1	75.5±11.0	76.7±10.4	77.3±10.0	78.1±9.8	<0.0001
Left bundle branch block, n (%)	16,719	814 (4.9)	107 (9.6)	169 (6.9)	399 (4.5)	139 (3.3)	<0.0001
LVEF, mean (SD), %	15,998	55.6±11.2	52.0±12.9	54.8±11.8	56.1±10.9	56.0±10.5	<0.0001
Fasting blood glucose, median (Q1, Q3), mmol/L	19,811	5.7 (5.1, 6.7)	5.9 (5.1, 7.3)	5.8 (5.2, 6.7)	5.6 (5.1, 6.5)	5.7 (5.1, 6.7)	<0.0001
HbA_1C_, mean (SD), %	6,747	6.8 (1.9)	7.1 (1.6)	6.9 (2.4)	6.7 (1.6)	6.9 (2.0)	<0.0001
Total cholesterol, median (Q1, Q3), mmol/L	21,185	4.3 (3.6, 5.0)	4.2 (3.5, 4.9)	4.3 (3.6, 5.1)	4.3 (3.6, 5.0)	4.2 (3.6, 5.0)	0.002
High-density lipoprotein	18,839	1.1 (1.0, 1.4)	1.1 (0.9, 1.3)	1.1 (1.0, 1.4)	1.1 (1.0, 1.4)	1.1 (0.9, 1.3)	<0.0001
Low-density lipoprotein	17,812	2.3 (1.9, 2.9)	2.3 (1.8, 2.9)	2.3 (1.8, 2.9)	2.4 (1.9, 2.9)	2.4 (1.8, 3.0)	0.0012
Fasting triglycerides, median (Q1, Q3), mmol/L	19,586	1.4 (1.0, 2.0)	1.5 (1.1, 2.1)	1.4 (1.1, 2.0)	1.4 (1.0, 1.9)	1.4 (1.0, 2.0)	<0.0001
Haemoglobin, median (Q1, Q3), mmol/L	18,714	8.7 (8.1, 9.3)	8.0 (7.2, 8.7)	8.5 (7.8, 9.1)	8.8 (8.2, 9.4)	8.9 (8.3, 9.4)	<0.0001

Abbreviations: BP, blood pressure; CKD-EPI, Chronic Kidney Disease–Epidemiology Collaboration; eGFR, estimated glomerular filtration rate; LVEF, left ventricular ejection fraction; SD, standard deviation.

a *p*-value tests for differences across eGFR groups.

Drug therapy is detailed in Table 3. High use of secondary prevention was seen across all CKD stages (overall 93.4% for lipid-lowering drugs, 95.3% antiplatelets, 75.9% beta-blockers). When considering the whole study population, statins were being taken by 84.3% of patients; a small but significant stepwise reduction in use was seen in patients with lower eGFR. Antiplatelet use was progressively reduced in patients with lower eGFR; the converse was seen for oral anticoagulant use. When considering patients with a history of AF, the proportion receiving oral anticoagulation significantly increased in a step-wise fashion as CKD class deteriorated. The use of calcium channel antagonists, ARBs, diuretics, digoxin, amiodarone and insulin was increased in those patients with lower eGFR. ACE inhibitor use was lower and inversely related to lower eGFR. A similar pattern of use was seen for patients (n = 13,759) with a history of MI (data not shown).

**Table 3:** Drug treatment for all patients with available data according to eGFR group calculated using the CKD-EPI.

Variable	Total (*n* = 22,272)	eGFR (mL/min/1.73 m^2^)				*p*-value^a^
		<45 (*n* = 1,570)	45–59 (*n* = 3,443)	60–89 (*n* = 11,958)	≥90 (*n* = 5,589)	
Antiplatelet, *n* (%)	21,228 (95.3)	1,385 (90.6)	3,152 (93.0)	11,288 (95.5)	5,403 (97.7)	<0.0001
Aspirin, *n* (%)	19,876 (89.3)	1,238 (81.1)	2,900 (85.6)	10,578 (89.5)	5,160 (93.4)	<0.0001
Thienopyridine, *n* (%)	5,707 (25.7)	404 (26.5)	797 (23.6)	2,872 (24.3)	1,634 (29.6)	<0.0001
Other antiplatelet, *n* (%)	2,067 (9.3)	153 (10.0)	299 (8.8)	1,050 (8.9)	565 (10.2)	0.02
Oral anticoagulant, *n* (%)	1,734 (7.8)	205 (13.5)	353 (10.4)	890 (7.5)	286 (5.2)	<0.0001
Oral anticoagulants in AF^b^, *n* (%)	753 (43.9)	110 (51.2)	192 (46.5)	374 (42.4)	77 (37.7)	0.02
Neither antiplatelet nor oral anticoagulant, *n* (%)	399 (1.8)	35 (2.3)	81 (2.4)	216 (1.8)	67 (1.2)	0.0002
Beta-blocker, *n* (%)	16,906 (75.9)	1,160 (75.9)	2548 (75.2)	8,861 (74.9)	4,337 (78.5)	<0.0001
Ivabradine, *n* (%)	2,177 (9.8)	150 (9.8)	362 (10.7)	1,162 (9.8)	503 (9.1)	0.11
Calcium antagonist, *n* (%)	6,069 (27.3)	513 (33.6)	1,076 (31.7)	3,204 (27.1)	1,276 (23.1)	<0.0001
Verapamil or diltiazem, *n* (%)	1,287 (5.8)	86 (5.6)	196 (5.8)	720 (6.1)	285 (5.2)	0.11
Dihydropyridine, *n* (%)	4,827 (21.7)	431 (28.2)	884 (26.1)	2,509 (21.2)	1,003 (18.1)	<0.0001
ACE inhibitor, *n* (%)	11,586 (52.0)	676 (44.2)	1,715 (50.6)	6,190 (52.3)	3,005 (54.4)	<0.0001
ARB, *n* (%)	5,951 (26.7)	570 (37.3)	1,086 (32.1)	3,086 (26.1)	1,209 (21.9)	<0.0001
Neither ACE nor ARB, *n* (%)	5,169 (23.2)	342 (22.4)	674 (19.9)	2,763 (23.4)	1,390 (25.1)	<0.0001
Lipid lowering, *n* (%)	20,791 (93.4)	1,412 (92.4)	3,110 (91.8)	11,054 (93.5)	5,215 (94.3)	<0.0001
Statin, *n* (%)	18,776 (84.3)	1,251 (81.9)	2,783 (82.1)	10,033 (84.8)	4,709 (85.2)	<0.0001
Diuretic, *n* (%)	6,614 (29.7)	836 (54.7)	1,399 (41.3)	3233 (27.3)	1,146 (20.7)	<0.0001
Other antihypertensive drug, *n* (%)	1,660 (7.5)	245 (16.0)	303 (8.9)	790 (6.7)	322 (5.8)	<0.0001
Digoxin and derivatives, *n* (%)	599 (2.7)	94 (6.2)	141 (4.2)	279 (2.4)	85 (1.5)	<0.0001
Amiodarone/dronedarone, *n* (%)	632 (2.8)	90 (5.9)	179 (5.3)	289 (2.4)	74 (1.3)	<0.0001
NSAID, *n* (%)	1,186 (5.3)	94 (6.2)	217 (6.4)	659 (5.6)	216 (3.9)	<0.0001
Insulin, *n* (%)	1,533 (6.9)	245 (16.0)	283 (8.4)	656 (5.5)	349 (6.3)	<0.0001
Oral antidiabetic agent, *n* (%)	4,833 (21.7)	425 (27.8)	813 (24.0)	2,364 (20.0)	1,231 (22.3)	<0.0001
All secondary preventive measures, n (%)	13,936 (62.6)	893 (58.4)	2,110 (62.3)	7,465 (63.1)	3,468 (62.7)	0.0049

Abbreviations: ACE, angiotensin converting enzyme; AF, atrial fibrillation; ARB, angiotensin receptor blocker; CKD-EPI, Chronic Kidney Disease–Epidemiology Collaboration; eGFR, estimated glomerular filtration rate; NSAID, non-steroidal anti-inflammatory drug.

a *p*-value tests for differences across eGFR groups.

b For patients with history of (or currently in) AF.

In the overall study population, 62.6% received all three secondary prevention treatments (antiplatelet plus statin plus ACE or ARB), with a significant reduction seen with lower eGFR (Table 3).

**Figure 2:**
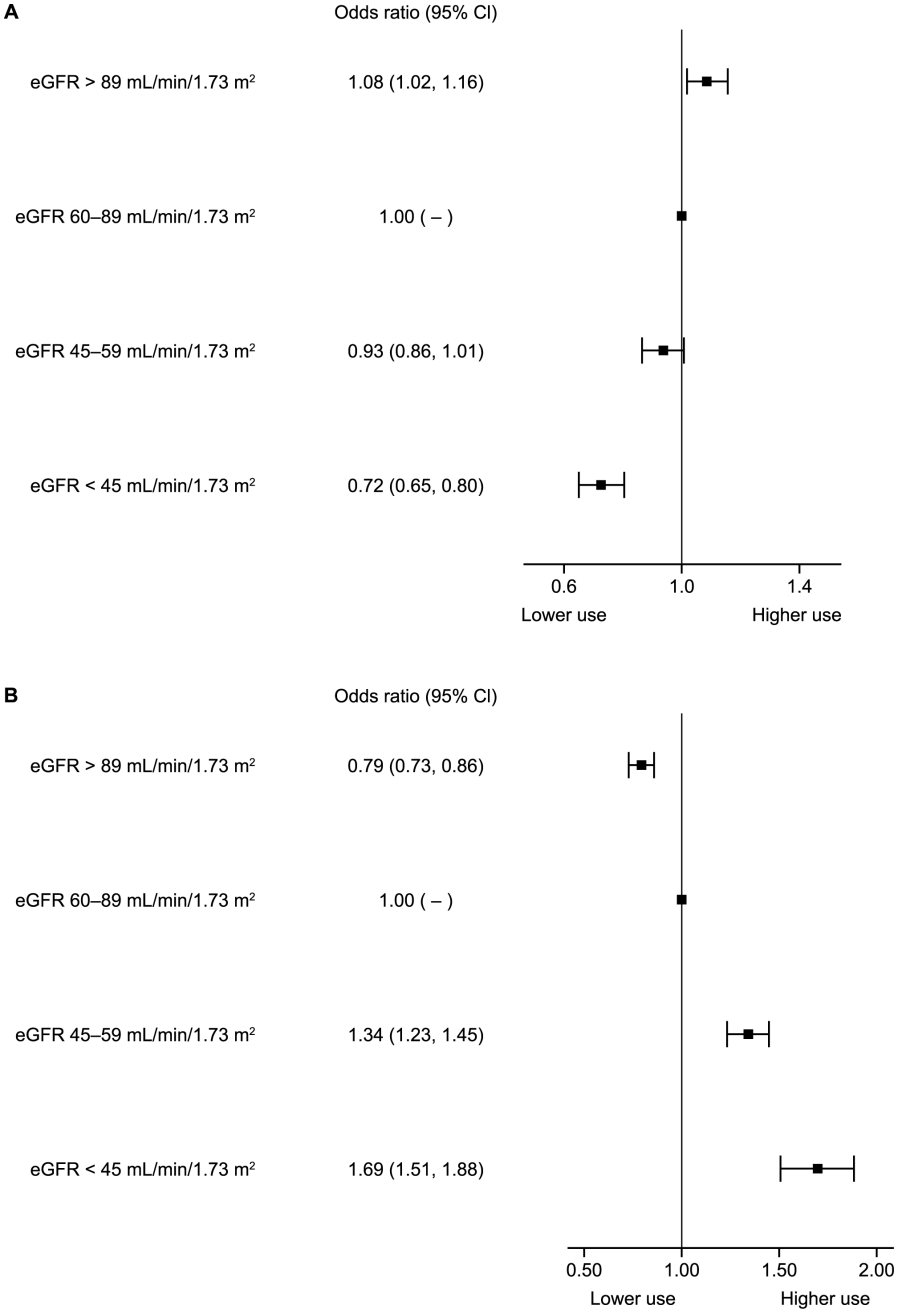
Univariate relationship between chronic kidney disease class and use of (A) angiotensin-converting enzyme inhibitors and (B) angiotensin receptor blocker.

**Figure 3:**
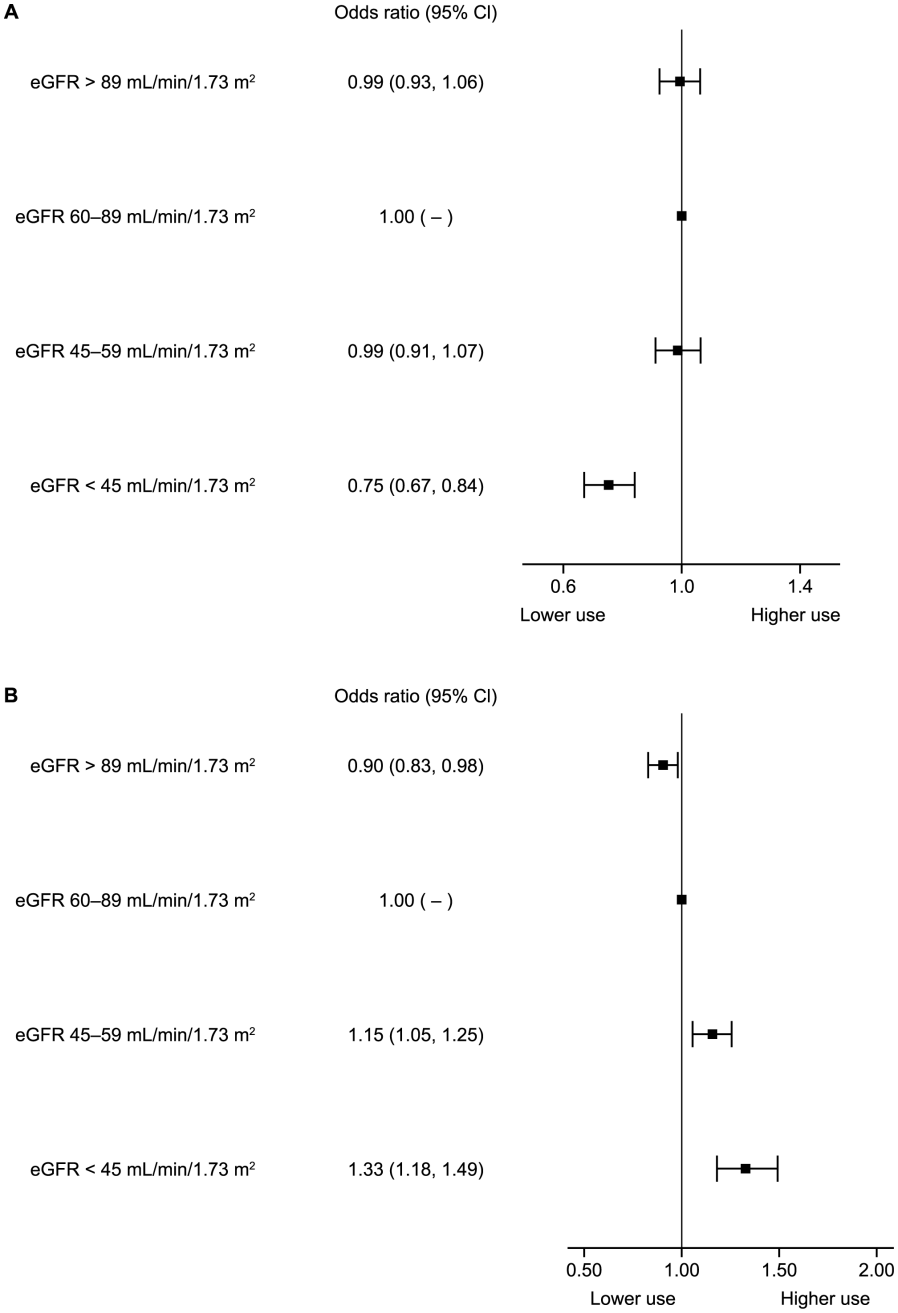
The association of use of (A) angiotensin-converting enzyme inhibitors and (B) angiotensin receptor blocker with chronic kidney disease following adjustment for age, body mass index, systolic blood pressure, diastolic blood pressure, gender, heart rate, smoking status, history of heart failure, angina, diabetes and hypertension.

We explored the relationship between renal function and the use of ACE/ARB further (Figures 2 and 3). Univariate analysis demonstrated that renal function (eGFR) was a significant and independent predictor for both ACE inhibitor and ARB use when considered as a continuous (data not shown) or categorical variable; lower eGFR values had reduced odds of ACE inhibitor use, whereas the converse was seen for ARB use. These associations were consistent following adjustment for a number of variables (age, body mass index, systolic blood pressure, diastolic blood pressure, gender, heart rate, smoking status, history of heart failure, angina, diabetes and hypertension). The odds of taking ACE inhibitors in the severely impaired renal function group were 0.75 (95% confidence interval [CI] 0.67, 0.84) compared to those with eGFR 60–89 mL/min/1.73 m^2^, whilst the odds of taking ARBs for the same group were 1.33 (95% CI 1.18, 1.49) compared to those with renal function 60–89 mL/min/1.73 m^2^. Patients with eGFR>90 mL/min/1.73 m^2^ had very similar results to those in the 60–89 mL/min/1.73 m^2^ group.

## Discussion

Published data on the prevalence of CKD in patients with stable CAD are scarce. The CLARIFY study population represents a unique, contemporary, global cohort of patients with stable CAD. In our study, where 22,272 patients were included, CKD (defined as eGFR <60 mL/min/1.73 m^2^) is seen in just under one-quarter of patients with CAD and is associated with increasing age, female sex, cardiovascular risk factors, overt vascular disease, other comorbidities, and higher systolic but lower diastolic blood pressure. Severe CKD (eGFR<45 mL/min/1.73 m^2^), which has previously been shown to be associated with poor prognosis, is as common as AF (6.9% and 7.3%, respectively).

Compared with recent published data, the prevalence of CKD in patients with CAD is greater than that seen in the general population. For example, contemporary data from a representative sample of the Canadian general population demonstrated a prevalence of CKD (any stage) of 12.5% [13]. The estimated prevalence of patients with CKD stages 3–5 (eGFR<59 mL/min/1.73 m^2^) was 3.1%, increasing to 18.6% when considering only subjects >65 years of age. The latter figure is perhaps more relevant, since the mean age of subjects in our study was 64 years. A recent study of consecutive patients receiving primary PCI for myocardial infarction in the UK demonstrated a mean age of approximately 64 years, with 17.6% exhibiting CKD stages 3–5 [14]. In the Canadian study [13], the prevalence of comorbidities such as hypertension and diabetes mellitus was greater in those with CKD, but no real difference in lipid profile (excepting triglycerides) was seen. A similar finding in relation to lipid profile was seen in our population, where no meaningful clinical difference in lipids levels was seen across the CKD stages. The United States National Health and Nutrition Examination Survey dataset show a total crude CKD prevalence estimate for adults aged ≥20 years to be 16.8%, of which 5.4% were in CKD stage 3 and 0.4% in stages 4 or 5 [15]. The prevalence of renal disease was significantly associated with age. The finding that almost one-quarter of subjects in the current study had CKD is likely to be explained in part by age but also by the high presence of comorbidities in patients with CAD, when compared with the general unselected population. For example, when considering all subjects, 30.4% had a history of diabetes mellitus, 71.9% treated hypertension, 75.4% dyslipidaemia and 61.8% prior myocardial infarction. Patients with severe CKD demonstrated a significantly higher prevalence of diabetes mellitus (45.0%) and hypertension (84.9%). Female sex was associated with lower eGFR; this relationship is likely to be influenced by increasing age and is in keeping with previous studies of patients with CAD [1].

It is not possible from the current study to determine if the presence of comorbid conditions led to the development of concomitant CAD and CKD (i.e. shared aetiological risk) or whether when present they contribute to accelerated decline in renal function. Alternatively renal impairment *per se* may contribute to adverse risk factor profile, such as hypertension. For example, the finding that wider pulse pressure, a marker of increased vascular stiffness [16], was more apparent in subjects with CKD could lead to the conclusion that it may underlie (at least in part) the renal dysfunction; yet it is equally plausible that it might be secondary to it (or perhaps both). It is hoped that the prospective nature of the CLARIFY study, with annual data collection, may help to tease out some of these complex relationships in more detail.

Secondary prevention was generally very good across all categories of renal function with no meaningful differences in use of beta-blockers. This is a major difference when the current data are compared with historical datasets, most of which were obtained in an acute or subacute care setting [8], [17]–[19]. These findings were consistent across subgroups of patients, such as those with a history of MI or CABG. A small but significant stepwise reduction in statin use was seen in patients with lower eGFR. Antiplatelet therapy use declined with lower eGFR; this may be explained by the concomitant increase in use of anticoagulant therapy and higher prevalence of AF with impaired renal function.

To try to identify any gap in implementation of secondary prevention in clinical practice, we assessed the number of patients within each renal function group who were taking evidence-based therapy in the form of an antiplatelet, a statin, and an ACE inhibitor or ARB. Just under two-thirds (62.6%) of the patients were found to be on this combination of drugs and a significant reduction was seen in patients with CKD (e.g. 58.4% in patients with CKD stage 3b–5). It is possible that the appropriate utilization of anticoagulant therapy for patients with AF may have influenced use of antiplatelet therapy.

Just over three-quarters (76.8%) of the overall patient group received either an ACE inhibitor or ARB (or both), and this order of magnitude was even seen when considering patients with severe CKD (stages 3b–5, 77.6%). However, ACE inhibitors were not used in a similar proportion across the eGFR groups; a step-wise reduction in use was seen in patients with lower eGFR. The opposite was seen for ARBs. These findings were independent of a number of clinical variables that might impact on use of these drugs, including history of hypertension, diabetes and age. A similar trend of higher use of ARBs following PCI for ST-elevation MI was seen in patients with severe CKD in a recently published UK study [14]. Guidelines relating to the management of patients with CKD do not differentiate between the use of ACE inhibitors or ARBs [20]. Further evaluation is required to fully understand these associations.

When considering patients with a history of AF, the proportion receiving oral anticoagulation significantly increased in step-wise fashion as CKD class deteriorated (37.8% amongst patients with CKD stage 1 to 51.2% in stages 3b–5). Renal impairment has recently been shown to be associated with higher risk of stroke and peripheral embolism in patients with AF [21].

Significant differences in the rates of use of other drug classes were seen across CKD stages. For example, dihydropyridine calcium channel blockers, diuretics, other antihypertensive drugs, digoxin and amiodarone were more frequently prescribed in patients with lower eGFR, most likely reflecting the associated high prevalence of comorbidities, including hypertension and AF.

This study has a number of limitations. For example, there are no data available with respect to albuminuria, and data on left ventricular function (e.g. from echocardiogram) were not systematically required for the study. The current study population comprises 68% of the total CLARIFY population, due to insufficient baseline data being available in the remainder. In some countries ethnicity data were not collected due to legal or ethical reasons (hence eGFR could not be calculated) and not all subjects had recent local laboratory data available (not mandated for the study). Whilst CKD *per se* was not an exclusion criterion, it is plausible that subjects with more severely impaired renal function may have been excluded on the basis of this being considered as hampering the collection of 5-year follow-up data. Renal function has been analysed according to eGFR, which is derived from a single serum creatinine assessment. This might lead to an overestimate of the prevalence of CKD stages 3–5. Whilst most western laboratories use a creatinine method that has calibration traceable to an isotope dilution mass spectrometry reference measurement procedure, given the international scope of this collaborative study it was difficult to establish the creatinine validation technique for all laboratories. It is plausible that this may have led to some error in attribution of diagnosis of CKD, but which was unavoidable. We chose to use CKD-EPI, as it performs better at higher GFRs, and data suggest that it may provide enhanced cardiovascular risk prediction compared with the Modification of Diet in Renal Disease (MDRD) method [21]. It is acknowledged that in clinical practice, decision-making regarding medical treatment by the responsible clinician is most likely to be influenced by serum creatinine and/or eGFR (e.g. in several countries MDRD-derived eGFR is routinely reported by laboratories). We believe that these potential limitations are overcome by the large size of the described cohort and the fact that the key findings are consistent irrespective of the method used to analyse renal function (in general qualitatively similar results with the MDRD formula [data not shown]). The data presented relate to ‘current treatments’ (i.e. those being taken when the patients were assessed at baseline). Owing to the nature of an observation study, it is not possible to ascertain whether patients were actually taking their prescribed medications.

In summary, baseline data from the CLARIFY study show that CKD is common in patients with stable CAD and is associated with age and comorbidities. Secondary prevention on the whole appears to be good, with high use of antiplatelet agents, statins, and beta-blockers. Around three-quarters of patients were prescribed either an ACE inhibitor or ARB, irrespective of renal function. In patients with severe CKD, there is lower use of ACE inhibitors, whereas ARBs are increasingly used. Yet the study shows opportunities for improvement in terms of increasing the proportion of patients taking all three evidence-based therapies for cardiovascular prevention. Many questions regarding the relationship between CKD and CVD remain unanswered. We believe that prospective data from the CLARIFY study will, in due course, help shed further light on this by permitting evaluation of the association of renal function on cardiovascular outcomes and mortality. The study will also assess the prognostic importance of and predictors for change in renal function over time.

## Supporting Information

Table S1 CLARIFY Registry Investigators.
(DOC)

Table S2 Chronic Kidney Disease–Epidemiology Collaboration formula.
(DOCX)
