# Supplementary material for: Impact of Chronic Kidney Disease on Use of Evidence-Based Therapy in Stable Coronary Artery Disease: A Prospective Analysis of 22,272 Patients
Source: PLoS One. 2014 Jul 22;9(7):e102335. doi: 10.1371/journal.pone.0102335 (PMC4106833; doi:10.1371/journal.pone.0102335)
Supplement: Table S2 — Chronic Kidney Disease–Epidemiology Collaboration formula. (DOCX) [file pone.0102335.s002.docx]

**Table S2.** Chronic Kidney Disease–Epidemiology Collaboration formula.

This equation is used to estimate glomerular filtration rate (GFR) and uses race, gender, age (years) and serum creatinine (mg/dL). It can be expressed as a single equation (adapted from Levey et al. 2009 [[1](#_ENREF_1)]) or be broken down as follows:

| Race | Gender | | Creatinine (mg/dL) | | Equation | |
| --- | --- | --- | --- | --- | --- | --- |
| Black | Female | ≤0.7 | | GFR = 166 × (S_cr_/0.7)^-0.329^ × (0.993)^Age^ | |  |
| Black | Female | >0.7 | | GFR = 166 × (S_cr_/0.7)^-1.209^ × (0.993)^Age^ | |  |
| Black | Male | ≤0.9 | | GFR = 163 × (S_cr_/0.9)^-0.411^ × (0.993)^Age^ | |  |
| Black | Male | >0.9 | | GFR = 163 × (S_cr_/0.9)^-1.209^ × (0.993)^Age^ | |  |
| White or other | Female | ≤0.7 | | GFR = 144 × (S_cr_/0.7)^-0.329^ × (0.993)^Age^ | |  |
| White or other | Female | >0.7 | | GFR = 144 × (S_cr_/0.7)^-1.209^ × (0.993)^Age^ | |  |
| White or other | Male | ≤0.9 | | GFR = 141 × (S_cr_/0.9)^-0.411^ × (0.993)^Age^ | |  |
| White or other | Male | >0.9 | | GFR = 141 × (S_cr_/0.9)^-1.209^ × (0.993)^Age^ | |  |

**Reference**

1. Levey AS, Stevens LA, Schmid CH, Zhang YL, Castro AF, 3rd, et al. (2009) A new equation to estimate glomerular filtration rate. Ann Intern Med 150: 604-612.
